# Intergroup biologization and outgroup prejudice in the time of COVID‐19

**DOI:** 10.1111/jasp.12831

**Published:** 2021-08-30

**Authors:** Roberta Rosa Valtorta, Cristina Baldissarri, Chiara Volpato, Luca Andrighetto

**Affiliations:** ^1^ Department of Psychology University of Milano‐Bicocca Milano Italy; ^2^ Department of Educational Sciences University of Genova Genova Italy

## Abstract

Through two studies (*N* = 602) conducted in Italy between February and March 2020, we examined the impact of the COVID‐19 emergency on biologization—a form of dehumanization that involves the perception of others as contagious entities—and outgroup prejudice. Overall, results showed that higher emergency perception was associated with greater biologization toward the groups most affected by the virus, namely the Chinese outgroup and the Italian ingroup. In turn, biologization toward the outgroup increased prejudice against that group. We also found that when the pandemic hit Italy, the greater emergency perception was associated with increased emotional closeness with Chinese people, resulting in reduced biologization and prejudice toward them. However, these results held true only for Italian respondents who reported higher levels of ingroup biologization. Taken together, our findings contribute to the knowledge gaps of biologization and prejudice by also providing relevant insights into the ongoing health emergency.

## 1 INTRODUCTION

A worldwide outbreak of the novel coronavirus SARS‐CoV‐2 causing COVID‐19 disease has begun in December 2019. At the time of writing this paper (August 3, 2021), COVID‐19 has infected over 199,644,978 people, claimed 4,250,237 lives worldwide, and continues to threaten humanity’s health and well‐being.^1^ Throughout history, infectious diseases have usually been associated with “othering,” that is, the reductive action of labeling and defining a person as an entity belonging to a socially subordinate category (White, 2020). In line with this evidence, following the spread of COVID‐19 from Wuhan, China, discrimination toward Chinese people has increased. This has included individual acts, such as microaggression or violence, and collective forms, such as barring Chinese people from establishments (Chung & Li, 2020). From a social‐psychological perspective, all these events can be explained in the light of the behavioral immune system theory, according to which human social interactions are likely to be shaped by pathogen stress (Murray & Schaller, 2016; Neuberg et al., 2011; Schaller & Park, 2011; Schnall, 2016).

Some of the most striking findings in the behavioral immune system literature suggest that pathogens—and human pathogen‐avoidance motives—might influence intergroup perceptions and behaviors. At the societal level, regions with high pathogen stress levels are more religious, collectivistic, and less trusting of outgroups (Fincher & Thornhill, 2012; Fincher et al., 2008; Zhang, 2018). Importantly, at the individual and intergroup level, studies suggest that participants exposed to pathogen cues report high ethnocentrism (Navarrete & Fessler, 2006; Navarrete et al., 2007), conformity (Wu & Chang, 2012), and negative perceptions toward outgroup members (Faulkner et al., 2004; Huang et al., 2011; Ji et al., 2019).

In line with the above evidence, research highlights the key role of pathogen‐avoidant responses and disease rhetoric in attitudes formation and in the stigmatization of those who appear diseased (Crandall & Moriarty, 1995; Schaller & Neuberg, 2012). In addition, by integrating evolutionary theory and metaphorical language’s effects, Brown and colleagues (2019) demonstrated how pathogen‐avoidant motives interact with figurative language to influence attitudes. More specifically, they found that the use of disease metaphors increases negative perceptions and anti‐immigration sentiments, especially among individuals with high levels of pathogen‐avoidance motivations.

Starting from these considerations, the present research aimed to extend the literature in this field by demonstrating that pathogen stress may elicit outgroup prejudice through specific dehumanizing perceptions. Indeed, as stated by Markel (1999), the threat of infectious diseases plays a crucial role in eliciting negative evaluations of others as a result of dehumanization against them. According to the author, during quarantine, the human beings who have been affected by the virus become the “enemy” in the same way as the virus itself. In this respect, the scholar argued that a common symptom of the quarantine mentality is to do everything possible to prevent the spread of an epidemic, often denying humanity to those who have encountered the disease and expressing negative feelings toward the potential sources of contagion.

Despite the relevance of this topic, to date, research has not provided clear evidence of the relationship between pathogen threat and dehumanizing representations. As a matter of fact, previous studies have mainly focused on the association between contagious diseases and prejudiced attitudes (e.g., Faulkner et al., 2004; Ji et al., 2019). To address this gap, through two studies conducted among Italian citizens in February 2020—when the COVID‐19 pandemic was not widespread in Italy—and in March 2020—when the pandemic had already broken out in this country—we intended to investigate the impact of the ongoing epidemiological situation caused by the novel coronavirus pandemic on outgroup prejudice via biologization, namely a dehumanizing process that involves the perception of individuals or groups as disease organisms (Douglas, 1966; Sontag, 2002; Volpato & Andrighetto, 2015).

## 2 PATHOGEN STRESS AND BIOLOGIZATION

Biologization is a form of dehumanization that employs metaphors linked to disease and has been theoretically examined within conflicting intergroup relations (Savage, 2007). In particular, several authors (e.g., Douglas, 1966; Hirsch & Smith, 1991; Sontag, 2002) have revealed that biological rhetoric has been widely used in the political domain and in relation to aggressive episodes. For example, along with all the other dehumanizing representations, Hitler’s *Mein Kampf* also included the conceptualization of Jews as harmful bacilli (Musolff, 2007). In a different context, Steuter and Wills (2010) analyzed the language used by Western mass media and revealed that biological metaphors such as cancer, metastasis, or viruses are often adopted for describing terrorist enemies. More recently, Moullagaliev and Khismatullina (2017) found that many conventional metaphors deployed in media discourse on migration derive from the area of “diagnosis of disease”, which frames outgroup members as dangerous health threats.

Crucially for the main goal of the present research, some studies have emphasized the key role of the salience of contagious diseases in people’s dehumanizing perceptions of others. For instance, Dalsklev and Kunst (2015) showed that exposing participants to a text focusing on the allegedly low hygienic standards of a minority group and their potential pathogen threat increased feelings of disgust and dehumanization, which, in turn, led to higher support of deportation. Importantly, Lawson et al. (2008) found that participants exposed to an editorial cartoon in which outgroup members were associated with disease subsequently dehumanized them more than participants who saw the same cartoon without reference to disease. Further, Valtorta, Baldissarri and colleagues (2019) focused on biologization in the work domain and found that the salience of dirty and unhealthy work environments characterizing certain low‐status occupations (i.e., garbage collectors and janitors) increased the association of these workers with biological metaphors. Overall, this literature suggests that biological dehumanization is triggered by categories of people who are perceived as more likely to carry pathogens, which pose an acute threat to well‐being or who are associated with specific types of infectious diseases. In other words, as reported by several authors (e.g., Dalsklev & Kunst, 2015; Esses et al., 2013), seeing individuals or groups as potential sources of disease transmission might be likely to lead to their dehumanization.

Building from these arguments, we assumed that the groups most affected by the novel coronavirus would be associated with an increase in dehumanizing perceptions in terms of biologization. More specifically, by integrating the abovementioned research on biological dehumanization (e.g., Savage, 2007; Valtorta, Baldissarri, et al., 2019) with that concerning the behavioral immune system theory (e.g., Murray & Schaller, 2016; Schaller & Park, 2011), we hypothesized that the perception of COVID‐19 emergency might lead to biologization toward the groups most afflicted by the virus. In addition, we assumed that biological dehumanization due to the health emergency should, in turn, be associated with increased prejudice. In the following section, we provide the rationale for our hypotheses.

## 3 DEHUMANIZATION AND PREJUDICE

A large number of studies (e.g., Dixon & Levine, 2012; Wilde et al., 2014) has shown that dehumanization and traditional prejudice are two different processes and dehumanizing an outgroup paves the way for negative treatments and prejudice toward that group (e.g., Costello & Hodson, 2010; Goff et al., 2008; Hodson & Costello, 2007). For example, Greenhalgh and Watt (2015) showed that Australians’ perception of value dissimilarity with refugees and asylum seekers was associated with greater prejudice toward them, and that this effect was mediated by dehumanizing perceptions. These findings echo those of Esses and colleagues (2008), who found that individuals who are higher in social dominance orientation, an ideology involving preferences for social hierarchy, were especially likely to dehumanize refugees by perceiving them as less characterized by human qualities of morality. As a result, they expressed more negative emotions and prejudice toward refugees. Thus, the authors demonstrated that once the process of dehumanization has begun, whether because of pre‐existing levels of social dominance orientation or due to information presented about a group, overall negative attitudes, prejudice, and a desire to exclude the group are likely to emerge. Furthermore, Costello and Hodson (2014) examined laypeople’s beliefs about the causes of and solutions to outgroup dehumanization and prejudice by concluding that dehumanization can be considered an important but largely unrecognized prejudice precursor.

Congruent with this previous research, we assumed that the emergency perception due to the COVID‐19 contagion would affect outgroup prejudice through increased dehumanizing representations. Importantly, as we will discuss below, we also assumed that the current COVID‐19 emergency would be associated with greater emotional closeness among the groups most impacted by the epidemic. In turn, such increased closeness should be associated with a decrease in the considered negative intergroup evaluations (i.e., biologization and prejudice).

## 4 HUMAN TRAGEDIES AND EMOTIONAL CLOSENESS

A growing body of research (e.g., Andrighetto et al., 2016; Eranen & Liebkind, 1993; Kaniasty & Norris, 2004) has demonstrated that peculiar circumstances, such as disaster exposure and human tragedies, may enhance prosocial attitudes and behaviors. In this regard, some scholars have written about the emergence of an “altruistic community” in the aftermath of hurricanes, floods, or earthquakes, characterized by high levels of solidarity and fellowship. For example, in research conducted in the United States after September 11, 2001, participants who reported higher suffering also reported emotional support, donating, and volunteering more than those who reported less suffering (Schuster et al., 2001). According to these results, Staub (2003, 2005) introduced the expression “altruism born of suffering” to indicate that individuals’ sufferings may enhance positive attitudes and motivation to help others. Several authors (e.g., Aron et al., 1992; Jacob et al., 2008; Vollhardt, 2009) argued that the “altruism born of suffering” could be explained by the fact that experiencing natural disasters may create the perception of a shared fate among individuals who suffered, or may change the relations with other individuals, such that one becomes emotionally closer to these others.

Moreover, as reported by Mawson (2007), acts of solidarity are often accompanied by feelings of closeness with people in the same situation. Relevant to the present research, the scholar argued that during natural disasters or other crises, affiliative behaviors are directed at people who shared the same psychological experiences. Thus, a state of heightened empathy and emotional closeness prevails, exemplified by increased mutual liking and “we‐feeling”. Consistently, Aron and colleagues (2004) stated that under some conditions such as human tragedies, when someone in the ingroup perceives to have something in common with an outgroup person, the effect is that, to some extent, ingroup members begin to see the outgroup as a part of their own group. Therefore, in these situations, the ingroup–outgroup distinction, vital to produce negative intergroup attitudes, is directly diminished by the outgroup member’s connection to an ingroup member. Furthermore, negative attitudes toward the outgroup are reduced and positive attitudes are enhanced by this closeness (and inclusion) of the outgroup with (in) the self.

Starting from these considerations, after examining the impact of the current health emergency on biologization and prejudice toward the group who first has encountered the novel coronavirus outbreak, namely Chinese people (Study 1), we aimed to investigate the potential role of emotional closeness in reducing negative perceptions against this outgroup (Study 2). In particular, we assumed that the ongoing COVID‐19 emergency in Italy would be associated with greater Italians’ emotional closeness with Chinese people. In turn, such increased closeness should be associated with decreased biologization and outgroup prejudice. Importantly, we investigated whether this pattern emerges specifically among Italian participants who reported higher levels of ingroup biologization. Indeed, Bastian and Crimston (2014) have demonstrated that people who experience self‐dehumanization in response to exceptional situations are more likely to report positive evaluations and engage in prosocial behaviors. In line with these findings, Bastian and colleagues (2013; see also Jordon et al., 2011; Zhong & Liljenquist, 2006) stated that when people feel that they have lost their humanity, they may be motivated to engage in a positive or prosocial way with others, thereby reconnecting them back into their human community and re‐establishing their moral status. Thus, in some circumstances, self‐perceiving as losing one’s own humanity may be an important step toward ending—rather than perpetuating—the cycle of inhuman behaviors. In this sense, it is plausible to think that experiencing higher ingroup biologization would lead Italian participants to report emotional closeness and positive responses toward Chinese people, in an effort to regain the lost humanity. In addition, it is possible that people who dehumanize Italians and Chinese might perceive these groups as more akin to each other than people who do not dehumanize their ingroup. This similar perception might wash away part of the ingroup‐outgroup separation, by thus strengthening the associations among emotional closeness, dehumanization, and prejudice toward Chinese people.

## 5 STUDY 1

Study 1 was conducted in February 2020, immediately after the announcement by the World Health Organization of the novel coronavirus pneumonia of China as a Public Health Emergency of International Concern and just before that this pneumonia massively targeted also the Italian population.

This study aimed to assess whether the Italians’ emergency perception due to the COVID‐19 contagion affected biologization and prejudice toward Chinese people, namely the group who first had to struggle with a massive outbreak of the virus (79,824 confirmed cases and 2,870 deaths as of February 29, 2020). More specifically, we assumed that the peculiar conditions characterizing the novel coronavirus epidemic would affect prejudice toward Chinese people via biological dehumanization. Indeed, as reported above, dehumanization is an extreme response to extraordinary situations that usually facilitates negative judgments and treatments against others. Furthermore, to verify the specificity of this link toward the Chinese outgroup, a comparison target group was included. In particular, we decided to consider North African people because, in our research context (i.e., Italy), this group has usually been subject to harshness and hostility (Caricati et al., 2017; Kirchler & Zani, 1995; Volpato & Durante, 2010). We supposed indeed that the health emergency period for epidemic contagion from COVID‐19 would promote prejudice via biological dehumanizing perceptions toward Chinese people but not against a negatively perceived group that is not affected by the epidemic to such a great extent (i.e., North African people; 2 confirmed cases and 0 deaths as of February 29, 2020).^2^

### 5.1 Method

#### 5.1.1 Participants and procedure

We recruited Italian participants using Prolific Academic, which allowed us to obtain data from a heterogeneous sample. Given the correlational nature of our study, we aimed at collecting data on a large sample (i.e., *N* > 250) that would guarantee the stability of the tested correlations (Schönbrodt & Perugini, 2013) and a power of 0.80 for correlation as low as 0.17, as determined by a priori power analysis conducted using G*Power (Faul et al., 2007). Therefore, we considered an initial sample of 326 respondents. In order to obtain a reliable sample of respondents and to identify inattentive respondents, we included two attentional check items in our survey (e.g., “Please answer 3 to this item”; see Oppenheimer et al., 2009). Twenty‐six participants failed these items and were removed from the analyses. Thus, the final sample was composed of 300 Italian participants (124 females, 173 males; *M*_age_ = 27.69, *SD* = 8.48; age range: 18–60).

#### 5.1.2 The survey

The following scales were used to measure participants’ perceptions during the emergency period due to the COVID‐19 spread. The order of presentation of the scales was randomly varied. The target groups to whom participants were asked to respond were Chinese people, North African people, and Germans. This last group was included as filler to mask the primary groups of interest: Chinese and North African people. After fulfilling the scales described below, participants were asked to indicate some demographic information about themselves. They were then debriefed and thanked for their participation.

##### Emergency perception

Participants rated their emergency perception by answering the question: “What level of emergency would you attribute to the current situation related to coronavirus?” on a scale from 0 (*low‐level emergency*) to 100 (*high‐level emergency*).

##### Biologization

Biologization was measured using seven disease‐related nouns (i.e., *disease, infection, virus*, *epidemic, contamination*, *filth*, and *contagion*; *α* for Chinese people = 0.97, *α* for North African people = 0.97, *α* for Germans = 0.94) borrowed from previous research (e.g., Valtorta, Baldissarri, et al., 2019; Valtorta & Volpato, 2018). Respondents were asked to rate the extent to which Chinese people, North African people, and Germans could be considered similar to these words by answering on a 7‐point Likert scale (1 = *not at all*; 7 = *extremely*) the following question: “In your opinion, how much [Chinese people] can be regarded as a [disease]?”.

##### Prejudice

Prejudice toward each of the target groups was assessed using the Subtle Prejudice subscale of the Pettigrew and Meertens’ Blatant and Subtle Prejudice Scale (1995), adapted for the Italian context by Manganelli Rattazzi and Volpato (2003). In particular, the subscale was constituted by 10 items (e.g., “[Chinese people] should not push themselves where they are not wanted”; *α* for Chinese people = 0.76, *α* for North African people = 0.84, *α* for Germans = 0.65), valuable on a 7‐point Likert scale (1 = *not at all*; 7 = *extremely*).^3^

### 5.2 Results

#### 5.2.1 Introductory analyses

To compare the biologization and prejudice score across the considered groups, we performed two repeated measures ANOVAs (group: Chinese people, North African people, Germans).

Regarding biological dehumanization, Mauchly’s test indicated that the assumption of sphericity had been violated, *χ* ^2^(2) = 28.94, *p* < .001; therefore, degrees of freedom were corrected using Huynh‐Feldt estimates of sphericity (*ε* = 0.92). The results showed a main effect of group, *F*(1.84, 550.54) = 54.67, *p* < .001, $\eta_{p}^{2}$ = 0.16, indicating that participants biologized more Chinese and North African people than Germans, all *p*s < .001. Furthermore, despite a non‐significant difference (*p* = .156), a trend in the results showed that participants biologized more Chinese than North African people (see Table 1).

**TABLE 1:** Descriptive statistics and correlations among variables, Study 1

Variables	*Mean (SD)*	1	2	3	4	5	6
1. Emergency perception	46.21 (23.34)	–					
2. Biologization (C)	1.81 (1.31)	0.25**	–				
3. Biologization (NA)	1.72 (1.20)	0.13*	0.80**	–			
4. Biologization (G)	1.29 (0.63)	0.05	0.62**	0.63**	–		
5. Prejudice (C)	3.49 (0.92)	0.02	0.40**	0.32**	0.18**	–	
6. Prejudice (NA)	3.67 (1.07)	0.04	0.39**	0.40**	0.20**	0.66**	–
7. Prejudice (G)	2.99 (0.77)	−0.03	0.18**	0.20**	0.18**	0.57**	0.48**

Abbreviations: C, Chinese people; G, Germans; NA, North African people.

* *p* ≤ .05

** *p* ≤ .001.

For prejudice, Mauchly’s test indicated that the assumption of sphericity had been violated, *χ* ^2^(2) = 21.58, *p* < .001; therefore, degrees of freedom were corrected using Huynh‐Feldt estimates of sphericity (*ε* = 0.94). The analysis showed a main effect of group, *F*(1.88, 562.34) = 99.34, *p* < .001, $\eta_{p}^{2}$ = 0.25: participants exhibited more prejudice toward Chinese and North African people than Germans, all *p*s < .001. Furthermore, the prejudice score toward North African people was higher than the score reported against Chinese people, *p* = .001 (see Table 1).

As shown in Table 1, emergency perception was positively correlated with biologization toward both our target groups (i.e., Chinese and North African people), whereas it was unrelated to prejudice. Further, the prejudice score was positively associated with biological dehumanization.

#### 5.2.2 Main analyses

**FIGURE 1:**
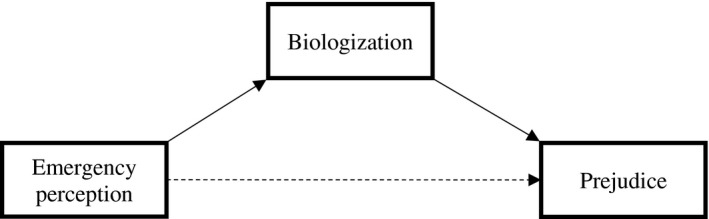
Schematic representation of the model tested in Study 1

To verify the prediction that the health emergency period would promote prejudice toward Chinese people (but not against North African people) via biologization, we tested two models using Hayes’ (2017) PROCESS macro (Model 4) and the bootstrapping method (5,000 resamples; see Figure 1).

As reported in Table 2, analyses indicated that health emergency perception was a significant predictor of biologization toward Chinese people. Moreover, biological dehumanization was positively associated with prejudice against this group. The direct effect of health emergency on prejudice was not significant; crucially, the proposed model was confirmed by the significance of the indirect effect via biologization, *a × b* = 0.01, *SE* = 0.01, 95% CI [0.01, 0.02].

**TABLE 2:** Regressions of emergency perception on prejudice toward Chinese people when biologization toward Chinese people is the mediator, Study 1

	*b* (*SE*)	*t*	95% CI		*p*
			Lower	Upper	
*Prediction of biologization*					
Emergency perception	0.01 (0.003)	4.39	0.01	0.02	<.001
*Prediction of prejudice*					
Emergency perception	−0.004 (0.002)	−1.66	−0.01	0.001	.098
Biologization	0.30 (0.04)	7.82	0.22	0.37	<.001

The same model was tested for North African people, and as reported in Table 3, analyses showed a similar pattern of results. Emergency perception increased biological dehumanization toward North African people. In turn, biologization was significantly related to an increase in prejudice. Despite the non‐significant direct effect, the indirect effect of emergency perception on prejudice via biologization emerged as significant, *a × b* = 0.003, *SE* = 0.001, 95% CI [0.0002, 0.005].

**TABLE 3:** Regressions of emergency perception on prejudice toward North African people when biologization toward North African people is the mediator, Study 1

	*b* (*SE*)	*t*	95% CI		*p*
			Lower	Upper	
*Prediction of biologization*					
Emergency perception	0.01 (0.003)	2.30	0.001	0.01	.022
*Prediction of prejudice*					
Emergency perception	−0.001 (0.003)	−0.27	−0.01	0.004	.784
Biologization	0.36 (0.05)	7.52	0.27	0.46	<.001

The present study showed that emergency perception due to the COVID‐19 spread was positively associated with biological dehumanization but not with outgroup prejudice. This finding is consistent with the literature (e.g., Dixon & Levine, 2012; Wilde et al., 2014), according to which dehumanization is distinct from traditional prejudice and is an extreme response to extraordinary situations. Crucially, our results revealed that higher levels of emergency perception increased biologization toward Chinese people that, in turn, affected prejudice against them. Contrary to our expectations, this pattern emerged also for North African people. This result could be explained by the fact that, in our research context, disease‐related metaphors are often used to describe immigrants and Black people (Volpato et al., 2010). In this sense, it is plausible to imagine that the salience of contagious disease due to the COVID‐19 epidemic may have reactivated the link between North African people and negative perceptions.

## 6 STUDY 2

This study aimed to further investigate the findings of Study 1 by adding the Italian ingroup as a new target group. Indeed, Study 2 was conducted in March 2020, when Italy became the first western country hit by the novel coronavirus (105,792 confirmed cases and 12,428 deaths as of March 31, 2020). In particular, given the exceptional epidemiological situation due to the COVID‐19 spread, through this study, we aimed to verify whether the Italians’ emergency perception was associated with biological dehumanization not only against Chinese people (81,554 confirmed cases and 3,312 deaths as of March 31, 2020) but also toward Italians themselves, namely the ingroup. As in the previous study, we considered North African people as the comparison target because this group was still one of the less affected by the epidemic on the date of the data collection (2,447 confirmed cases and 136 deaths as of March 31, 2020).^2^

Furthermore, in the light of the similar health emergency that both Chinese and Italian people struggled with at the time of the present study, we hypothesized that Italians perceived a higher emotional closeness with Chinese people (vs. North African people) and that this perception was negatively associated with biologization and prejudice toward this group. Indeed, according to several authors (e.g., Kofta & Slawuta, 2013; Lee & Kim, 2021) and as previously reported, psychological and emotional closeness in response to peculiar circumstances (e.g., natural disasters and human tragedies) are strongly related to positive behaviors and humanization of others.

In line with Study 1, we supposed that the emergency perception due to the COVID‐19 outbreak would affect prejudice toward Chinese people via biologization. In addition, we assumed that a health emergency would also promote emotional closeness between Italian participants and Chinese people. In turn, this perception would reduce biologization and prejudice toward this outgroup. Crucially, we investigated whether this pattern emerges specifically among Italian participants who reported higher levels of ingroup biologization.

### 6.1 Method

#### 6.1.1 Participants and procedure

Data were collected through a questionnaire using Qualtrics survey web‐system. A snowball sampling strategy was employed, with the initial participants recruited through the experimenters’ parental and friendship networks. In line with Schönbrodt and Perugini’s (2013) suggestions and Study 1, we recruited 330 Italian participants. As in the first study, we included two attentional check items in our survey. Twenty‐eight participants failed the attentional check questions and were removed from the analyses. Thus, the final sample was composed of 302 Italian participants (221 females, 81 males; *M*_age_ = 28.82, *SD* = 12.14; age range: 18–62).

#### 6.1.2 The survey

As in the previous study, the order of presentation of the following scales was randomly varied. The instructions of the questionnaire and some measures (i.e., biologization, *α* for Italians = 0.96, *α* for Chinese people = 0.97, *α* for North African people = 0.96; prejudice,^4^ *α* for Chinese people = 0.77, *α* for North African people = 0.82) were the same that were used in Study 1. However, in this study, participants were asked to think about Italians, Chinese, and North African people. Furthermore, unlike Study 1, a different measure of emergency perception and a scale of emotional closeness were included in the current study.

##### Emergency perception

Regarding emergency perception, we ad‐hoc created a more accurate six‐item scale (e.g., “The coronavirus emergency will affect my life also in the future”) instead of using the single‐item measure employed in Study 1. Participants were asked to express their agreement on a 7‐point Likert scale (1 = *not at all*; 7 = *extremely*). Scores were combined to yield an overall emergency perception score (*α* = 0.71); higher scores denote greater emergency perception due to the COVID‐19 disease.^5^

##### Emotional closeness

The Inclusion of Other in the Self (IOS; Aron et al., 1992) Scale was used to examine whether Italians perceived a higher overlap between their own self and Chinese people rather than between their own self and North African people. More specifically, participants were asked to choose among seven pictures the one that best represented their relationship in terms of emotional closeness with Chinese and North African people. Each image showed two circles (labeled “self” and “Chinese people” in one question, and “self” and “North African people” in the other) with varying degrees of overlap, from non‐overlapping (i.e., 1) to almost completely overlapping (i.e., 7).

After fulfilling the scales described above, participants were asked to indicate some demographic information about themselves. They were then debriefed and thanked for their participation.

### 6.2 Results

#### 6.2.1 Introductory analyses

To compare the biologization score across the considered groups, we performed a repeated‐measures ANOVA (group: Chinese people, North African people, Italians). Mauchly’s test indicated that the assumption of sphericity had been violated, *χ* ^2^(2) = 19.97, *p* < .001; therefore, degrees of freedom were corrected using Huynh‐Feldt estimates of sphericity (*ε* = 0.95). The results showed a main effect of group, *F*(1.89, 569.01) = 60.64, *p* < .001, $\eta_{p}^{2}$ = 0.17, indicating that the most biologized group was the ingroup one (i.e., Italians), all *p*s < .001. Furthermore, Chinese people were more biologized than North African people, *p* < .001 (see Table 4).

**TABLE 4:** Descriptive statistics and correlations among variables, Study 2

Variables	*Mean (SD)*	1	2	3	4	5	6	7
1. Emergency perception	4.25 (1.16)	‒						
2. Biologization (I)	3.26 (1.85)	0.22**	‒					
3. Biologization (C)	2.93 (1.91)	0.20*	0.76**	‒				
4. Biologization (NA)	2.31 (1.49)	0.05	0.54**	0.59**	‒			
5. Prejudice (C)	3.22 (0.89)	0.02	0.20**	0.29**	0.26**	‒		
6. Prejudice (NA)	3.35 (1.00)	0.02	0.18**	0.24**	0.35**	0.67**	‒	
7. Emotional closeness (C)	4.12 (1.73)	0.13*	−0.05	−0.11*	−0.14*	−0.43**	−0.32**	‒
8. Emotional closeness (NA)	3.53 (1.83)	0.04	−0.12*	−0.12*	−0.22**	−0.43**	−0.57**	0.61**

Abbreviations: C, Chinese people; I, Italians; NA, North African people.

* *p* ≤ .05

** *p* ≤ .001.

Two repeated‐measures ANOVAs (group: Chinese people, North African people) were then conducted to ascertain whether there were significant differences in prejudice and emotional closeness scores toward the two target groups.

As for prejudice, the analysis showed a significant effect of group, *F*(1, 301) = 7.91, *p* = .005, $\eta_{p}^{2}$ = 0.03, indicating that participants exhibited more prejudice against North African than Chinese people (see Table 4).

Regarding emotional closeness, in line with our assumption, results showed that Italian participants reported more closeness with Chinese people than with North African people, *F*(1, 301) = 42.76, *p* < .001, $\eta_{p}^{2}$ = 0.12 (see Table 4).

As shown in Table 4, the emergency perception was positively correlated with biologization toward both our target groups (i.e., Italians and Chinese people) but not with biological dehumanization against North African people. Moreover, in line with the results of Study 1, the emergency perception due to the COVID‐19 epidemic was unrelated to prejudice. Importantly, Italians’ emergency perception was positively associated with emotional closeness with Chinese people. This latter variable was negatively correlated with biologization and prejudice against Chinese people.

#### 6.2.2 Main analyses

**FIGURE 2:**
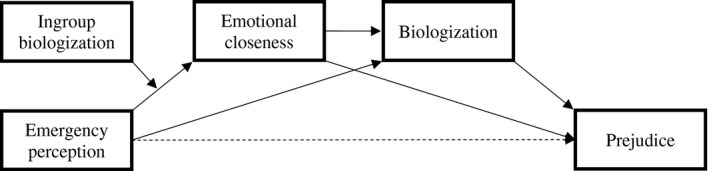
Schematic representation of the model tested in Study 2

To examine the role of ingroup biologization and the relationships among emergency perception, emotional closeness, and negative perceptions toward both Chinese and North African people, we tested two conditional process models. As reported in Figure 2, we considered participants’ emergency perception as the predictor variable, emotional closeness as the first‐level mediator, biologization as the second‐level mediator, and ingroup biologization as the moderator of the relationship between health emergency and emotional closeness. Finally, prejudice was entered as the outcome variable (Model 83 of the PROCESS macro for SPSS with 5,000 bootstrapping samples; Hayes, 2017).

As shown in Table 5, for the moderated path from emergency perception to emotional closeness with Chinese people, results showed a non‐significant effect of biologization toward Italians. Crucially, emergency perception and the two‐way interaction Emergency perception × Biologization toward Italians were positively associated with emotional closeness. As expected, decomposition of this interaction revealed that emergency perception was positively related with emotional closeness with Chinese people for Italian participants who reported higher levels of ingroup biologization, *b* = 0.44, *SE* = 0.13, *t*(298) = 3.37, *p* < .001, 95% CI [0.18, 0.70], whereas such a relation was not significant among participants who reported lower levels of ingroup biologization, *b* = 0.04, *SE* = 0.11, *t*(298) = 0.39, *p* = .697.

**TABLE 5:** Regressions of emergency perception on prejudice against Chinese people when emotional closeness with and biologization toward them are the first‐ and second‐level mediators and biologization toward Italians the moderator, Study 2

	*b* (*SE*)	*t*	95% CI		*p*
			Lower	Upper	
*Prediction of emotional closeness (C)*					
Emergency perception	0.24 (0.09)	2.78	0.07	0.42	.006
Biologization (I)	−0.09 (0.05)	−1.65	−0.20	0.02	.099
Emergency perception × Biologization (I)	0.11 (0.05)	2.30	0.02	0.20	.022
*Prediction of biologization (C)*					
Emergency perception	0.36 (0.09)	3.87	0.18	0.54	<.001
Emotional closeness	−0.16 (0.06)	−2.51	−0.28	−0.03	.013
*Prediction of prejudice (C)*					
Emergency perception	0.02 (0.04)	0.46	−0.60	0.10	.645
Emotional closeness	−0.21 (0.03)	−7.85	−0.26	−0.16	<.001
Biologization (C)	0.11 (0.02)	4.64	0.06	0.16	<.001

Abbreviations: C, Chinese people; I, Italians.

For the paths from the first‐level mediator to second‐level mediator and prejudice, we found that emotional closeness with Chinese people was related to decreased biological dehumanization and prejudice toward them. Most importantly, the conditional indirect effect of emergency perception on reduced prejudice toward Chinese people through greater emotional closeness was significant for Italian respondents who reported higher levels of ingroup biologization, *a × b* = −0.09, *SE* = 0.03, 95% CI [−0.15, −0.03], but not for Italian respondents who reported lower levels of ingroup biologization, *a × b* = −0.01, *SE* = 0.02, 95% CI [−0.06, 0.04]. Finally, in line with Study 1, also the indirect effect of health emergency via biologization toward Chinese people on prejudice was significant, *a × b* = 0.04, *SE* = 0.01, 95% CI [0.02, 0.07]. Therefore, the tested model was supported, as confirmed by the index of moderated mediation (IMM) = −0.002, *SE* = 0.002, 95% CI [−0.006, −0.001].

As for North African people, in line with our expectations, health emergency did not predict emotional closeness (*b* = 0.12, *p* = .189) and biological dehumanization (*b* = 0.07, *p* = .304). In the same vein, the two‐way interaction Emergency perception ×Biologization toward Italians was not associated with emotional closeness (*b* = 0.07, *p* = .151). Thus, the tested model was not supported.

As found in Study 1, results showed that the emergency perception due to the COVID‐19 spread was positively associated with biological dehumanization but not with outgroup prejudice. It is noteworthy that this pattern emerged not only when participants assessed Chinese people but also when Italians were asked to evaluate their own group. Importantly, in line with the previous study, our results revealed that higher levels of emergency perception increased biologization toward Chinese people (but not North African people) that in turn increased prejudice against them. In addition, we found that because of the similar health emergency that both Chinese and Italian people struggled with, this latter group perceived a higher emotional closeness with Chinese people, and this perception, in turn, reduced negative evaluations against them. However, this pattern of results emerged only for Italian participants that tended to perceive their own group in biological terms.

## 7 GENERAL DISCUSSION

The main aim of this research was to investigate the intergroup consequences of the epidemiological situation due to the spread of the novel coronavirus. Through two studies that considered Italian citizens between February and March 2020—when the epidemic was announced, and the number of infections rapidly increased in several countries—we demonstrated that the health emergency perception due to the COVID‐19 outbreak shaped the social perception of the involved groups in terms of biological dehumanization and outgroup prejudice. More specifically, in line with our assumptions, Study 1 revealed that higher levels of emergency perception among Italian participants increased biologization toward Chinese people that in turn affected prejudice against them. Despite this relevant finding, it is important to note that Study 1 showed a non‐significant difference between the levels of biological dehumanization reported toward Chinese people and toward the comparison target group that we included in the study, namely North African people. In addition, the same pattern of relationships among emergency perception, biologization, and prejudice that emerged for Chinese people was also found for North African people. These unexpected results can be explained in the light of the literature on dehumanizing perceptions and prejudice, according to which linguistic metaphors related to the disease are often used to describe the negative impact of immigrants on Italian society by thus increasing negative views of foreigners and outgroup members. Several research projects (e.g., European Commission against Racism and Intolerance (ECRI), 2016; Valtorta, Signorato, et al., 2019; Volpato et al., 2010) indicated indeed a widespread perception among the Italian population that immigrants and Black people represent a threat to security and well‐being. In this sense, it is plausible to think that the salience of contagious disease due to the initial phase of the COVID‐19 spread may have reactivated the link between North African people, dehumanization, and prejudice, even if this group was one of the less affected by the epidemic on the date of the data collection.

The impact of the COVID‐19 emergency on several negative outcomes in the field of intergroup relations was further investigated in Study 2, in which the first western population hit by the novel coronavirus, namely Italians, was added as a new target group. In particular, Study 2 showed a significant association between the health emergency perception and biological dehumanization addressed to the groups most affected by the contagion when the study was conducted (i.e., Italians and Chinese people versus North African people). In this regard, it is noteworthy that Italians were more biologized than Chinese people and North African people. In other words, participants reported more dehumanizing perceptions toward their ingroup than toward outgroups. This result can be interpreted as further evidence of the key role played by the current peculiar health emergency circumstances in eliciting biologization, also going beyond the traditional research on the intergroup dehumanization dynamics (e.g., Costello & Hodson, 2014; Leyens et al., 2000), which so far conceived dehumanization and its different forms especially as a means of denigrating outgroup members. In this regard, we believe that our findings complement research about self‐dehumanization. For example, the internalization of dehumanizing acts and perceptions emerged in the analysis conducted by Volpato and Contarello (1999) of Primo Levi’s *If This Is a Man* (1958), one of the most valuable testimonies of the Holocaust. According to Levi, dehumanization affected both victims and oppressors. In Levi’s eyes, “The personages in these pages are not men. Their humanity is buried, or they themselves have buried it” (p. 127). As reported by the authors, the text contains a great deal of self‐dehumanizing metaphors, especially in terms of animal imagery. The most exhausted prisoners remind Levi of “sled‐dogs in London’s books, who slave until the last breath and die on the track” (p. 49). On the other hand, the toughest prisoners have “the rudimentary astuteness of a draught‐horse, which stops pulling a little before it reaches exhaustion” (p. 48). In addition, one set of studies conducted by Bastian and Haslam (2010) linked experiences of social exclusion with self‐dehumanization by revealing that social exclusion heightened targets’ viewing themselves as having fewer human characteristics. Different factors (e.g., human tragedies, experiences of social exclusion) can therefore promote the internalization of dehumanizing traits by thus modifying how we see and perceive ourselves. The present research adds a tile to this picture by demonstrating that in peculiar circumstances such as an epidemic, biological dehumanizing representations can be adopted to describe one’s own group membership.

Relevant to our findings, several authors (e.g., Hu et al., 2018; Wu et al., 2015, 2019) suggested that the mechanism of ingroup derogation is related to the evolutive response of the behavioral immune system, and it is specifically triggered when dealing with a peculiar ecological condition in which greater threat of diseases is incurred by ingroup members. In this regard, studies (see Fincher & Thornill, 2012) have shown that, in some areas (e.g., Africa), the correlations between parasite stress and ingroup sociality were reported to be negative rather than positive. Overall, it seems robust that the pathogen stress significantly shapes attitudes and ingroup perceptions. For the first time in the literature, we provided preliminary evidence of this association by considering one of the most derogating form of social perception, namely dehumanization.

According to Study 1, results of Study 2 revealed that higher levels of emergency perception increased biologization toward Chinese people (but not toward North African people), which in turn increased prejudice and negative attitudes against them. In line with these findings, previous literature (e.g., see Schaller et al., 2015) revealed that emergency situations in which individuals are—or merely perceive themselves to be—more vulnerable because of a disease‐related condition, as the current pandemic, heighten protective behaviors by thus triggering stigmatization of others. As stated by Clissold and colleagues (2020), emergency situations characterized by outbreaks of infectious disease represent a fertile breeding ground for unveiling existing negative perceptions which are often driven by collective fear. Indeed, infectious disease and its correlates are seen as a threat, and therefore attempts are made to “other” this threat (Nelkin & Gilman, 1988; Reny & Barreto, 2020). These attempts at “othering” are commonly reinforced by a desire to assign blame and responsibility for the dangerous situation to make sense of such adversity. Evidence of these speculations was provided by Washer (2004), who conducted a study looking at the British media’s response to the 2002 SARS outbreak. The scholar found the development of a discourse suggesting that British citizens were protected from SARS and its detrimental consequences because they were “different” from the Asian citizens, who were initially affected by such an outbreak. Our results seem to be consistent with this literature and demonstrate that the current health emergency together with all its repercussions have a propensity to incite negative intergroup attitudes and perceptions.

Furthermore, we found that Italian participants perceived higher emotional closeness with Chinese people (vs. North African people). Importantly, this emotional closeness reduced biologization and prejudice toward this outgroup. Therefore, by expanding the behavioral immune system literature (e.g., Murray & Schaller, 2016; Schaller & Park, 2011), not only did we confirm the relationship between the salience of contagious disease and negative perceptions against outgroup members, but we also demonstrated that sharing the experience of a viral epidemic could reduce the negative effects of the pathogen stress via the increased emotional closeness among the involved groups. In this regard, it is important to note that this pattern of results emerged only among Italian respondents who reported higher levels of ingroup biologization. Of particular relevance to these findings, several studies (for a review, see Bastian & Crimston, 2014) provided an interesting twist on dehumanization by showing that dehumanizing the self or the ingroup in response to unethical behaviors or exceptional situations motivates a tendency to report and engage in prosocial responses. It is plausible to think that the underlying motivation to report positive attitudes toward the outgroup may represent an attempt to restore humanity within self‐perception. Possessing humanity is indeed a basic feature for our identity, and when it is lost, this would be expected to motivate attempts to restore it. In the case of the present research, the perception of “being the virus” because of the COVID‐19 epidemic, and the resulting self‐biologization, may have led Italian participants to report positive responses toward Chinese people (i.e., decrease in their biologization and prejudice) in an attempt to regain humanity lost.

Through the present studies, by integrating previous empirical findings concerning the behavioral immune system theory (e.g., Murray & Schaller, 2016; Park et al., 2007; Thornhill & Fincher, 2014) with the theoretical insights regarding biological dehumanization and prejudice (e.g., Costello & Hodson, 2010; Savage, 2007; Volpato & Andrighetto, 2015), we demonstrated that the exceptional epidemiological situation due to the COVID‐19 spread has the capacity to directly foster social‐cognitive biologization of both outgroup and ingroup, but not other forms of outgroup prejudice. In line with these findings, Wilde and colleagues (2014) analyzed dehumanizing processes by distinguishing them from negative attitudes and evaluations. In particular, the authors suggested that dehumanization is a distinct category rather than simply an extreme form of prejudice. Furthermore, a large number of studies (Costello & Hodson, 2010; Goff et al., 2008; Hodson & Costello, 2007; Leyens et al., 2000) have demonstrated that outgroup dehumanization can be considered a robust predictor of prejudice in intergroup contexts. These considerations are particularly relevant for the results that emerged in the present studies, according to which the health emergency perception affected prejudice toward outgroups via biological dehumanization. Our findings seem to confirm previous research on the crucial role of dehumanizing perceptions in shaping outgroup prejudice (e.g., Costello & Hodson, 2012; Goff et al., 2008), by also providing the first evidence of this relationship in such an exceptional situation as a pandemic.

It is noteworthy that although several researchers demonstrated that increased outgroup prejudice may stem from pathogen threat, some recent works showed discrepant findings regarding this specific link. For example, results by van Leeuwen and Peterson (2018) were inconsistent with the view that the behavioral immune system motivates outgroup prejudice. Instead, their findings suggested that this system “simply” motivates the avoidance of any infected individual, regardless of their group membership. In this sense, biologization could be considered a relevant process in explaining the differences concerning the literature on this topic. Indeed, our results are consistent with some scholars who speculated on the relationship between the behavioral immune system theory and dehumanization. For example, Prażmo and Augustyn (2020) stated that the metaphorical notion of a social and bio parasite to describe immigrants is strongly related to the activation of the behavioral immune system, whose aim is to protect us from having any other close contact with potentially contaminated. According to the authors, it may also affect our reasoning and decision‐making in relation to political inclinations and attitudes toward others. Thus, activating the parasite imagery inevitably leads to elicit defensive reactions and negative perceptions.

Finally, it is noteworthy that we found a non‐significant difference between biologization of Chinese and North African people in Study 1, whereas results of Study 2 showed that the North African group was significantly less biologized than the Chinese one. Furthermore, biologization of both the target groups was higher in Study 2 than in the first study (see Table 1 and Table 4). In other words, when Italy became the first western country hit by the novel coronavirus and Study 2 was conducted (i.e., March 2020), biologization became more severe, and we found a higher biologization score for Chinese than North African people. In this regard, it is plausible to think that Italian participants were still not quite as concerned in February 2020. Thus, as the pandemic progressed and broke out in this country, Italians reported a higher emergency perception and greater biological dehumanization of the groups most affected by the virus (i.e., Italians and Chinese people vs. North African people). As a matter of fact, we performed three independent samples *t*‐tests to compare the biologization scores toward Chinese and North African people and the emergency perception ratings reported in Study 1 and Study 2.^6^ Regarding both the target groups, results showed a significant difference, indicating that biologization was significantly higher in March 2020 (*M*_CP_ = 2.93, *SD*_CP_ = 1.91; *M*_NA_ = 2.31; *SD*_NA_ = 1.49) than in February 2020 (*M*_CP_ = 1.81, *SD*_CP_ = 1.31; *M*_NA_ = 1.72; *SD*_NA_ = 1.20), *t*(600) = −8.39, *p* < .001 for Chinese people; *t*(600) = −5.35, *p* < .001 for North African people. Crucially, in line with our speculations, we found that the emergency perception of Italian participants was significantly higher in March 2020 (*M* = 4.25, *SD* = 1.16) than in February 2020 (*M* = 3.77; *SD* = 1.40), *t*(600) = −4.58, *p* < .001.

## 8 LIMITATIONS AND FUTURE DIRECTIONS

Despite the novelty of our studies, it is important to acknowledge that our research has some limitations that may restrict its generalizability. Although the associations we observed among variables are consistent with previous findings on dehumanization and prejudice, the correlational nature of the current data does not allow us to draw any causal inferences. It is likely indeed that the relationships between some of our constructs are bidirectional and dynamic. Experimental or longitudinal studies would be an important next step toward determining the direction of these paths.

Moreover, it is important to note that in Study 1 and Study 2, participants were asked to think about Chinese and North African people, without providing other information about the groups. Therefore, it is not possible to know whether participants thought of the two target groups as immigrants in Italy or as people living in their respective countries. Given that the migration background might have been a confounding, further studies should deepen our findings by controlling for this aspect.

Finally, it is noteworthy that in both studies, the mean ratings of biological dehumanization were low, indicating a weak association of the targets with virus‐related words. However, it should be noted that our measure assessed the association between the target and dehumanized perceptions using a self‐report measure, which may have been affected by the participants’ desirability concerns (e.g., Crowne & Marlowe, 1964; Nederhof, 1985). Greater associations with biological metaphors may emerge in studies using a subtler measure of dehumanization and implicit techniques, which are less susceptible to motivated responding (Gawronski & Bodenhausen, 2006) and less explicitly related to the current pandemic. Related to this last issue, it is important to note that the indirect effects of the tested models were weak in both our studies. Further research is needed to corroborate our findings and the relationships among emergency perception, dehumanizing processes, and prejudicial attitudes.

## 9 CONCLUSIONS

Our findings reveal how being threatened with disease, as in the case of the COVID‐19 outbreak, is an important source of biologization. Crucially, we demonstrated the relevance of this dehumanizing perception in promoting prejudice against the potential sources of contagion. At the same time, we also showed that such negative effects could be reduced when ingroup members experience the same distressing situation, by virtue of the increasing emotional closeness among them. In addition, our results shed light on a new facet of biological dehumanization that, in such an exceptional situation, can become a relevant component not only for the way we see others but also for the way we perceive our own group and identity. We hope that this work will advance knowledge about biologization and prejudice and it will help us better understand the conditions that affect people’s perceptions toward both outgroup and ingroup members.

## CONFLICT OF INTEREST

The authors declare that there is no conflict of interest.

## Footnotes

^1^
All the statistics reported in the manuscript about confirmed cases and death toll due to the COVID‐19 disease were retrieved from the website http://worldometers.info/coronavirus/ (last accessed on August 3, 2021).

^2^
Confirmed cases and deaths in North Africa were computed by summing the statistics concerning Egypt, Tunisia, Morocco, Libya, and Algeria.

^3^
To further examine the relationships among the emergency perception due to the COVID‐19 disease, biologization, and outgroup prejudice, we measured this latter variable also by using the feeling thermometer (see Nelson, 2008). For both Study 1 and Study 2, analyses showed the same pattern of results emerged for the Subtle Prejudice scale (for more details, see the Supplementary Analyses document on the Open Science Framework, https://osf.io/a4mv2/?view_only=89823770ec0a4351bd5fa49928020b58).

^4^
Considering that we collected evaluations from an Italian sample, prejudice was measured only toward Chinese and North African people.

^5^
For the complete scale, see Supplementary Material on the Open Science Framework, https://osf.io/a4mv2/?view_only=89823770ec0a4351bd5fa49928020b58.

^6^
Given that we used different scales for emergency perception (from 0 to 100 in Study 1; from 1 to 7 in Study 2), before conducting the independent samples *t*‐test, we rescaled the measures to be on the same scale (i.e., 7‐point Likert scale; for more details, see the IBM SPSS Statistics support page on how to transform different scales to a common scale).
